# What Is the Performance of ChatGPT in Determining the Gender of Individuals Based on Their First and Last Names?

**DOI:** 10.2196/53656

**Published:** 2024-03-13

**Authors:** Paul Sebo

**Affiliations:** 1 University Institute for Primary Care University of Geneva Geneva Switzerland

**Keywords:** accuracy, artificial intelligence, AI, ChatGPT, gender, gender detection tool, misclassification, name, performance, gender detection, gender detection tools, inequalities, language model, NamSor, Gender API, Switzerland, physicians, gender bias, disparities, gender disparities, gender gap

## Introduction

Accurate determination of gender from names is vital for addressing gender-related disparities in medicine and promoting inclusivity. Gender detection tools (GDTs) offer efficient solutions, enabling large-scale demographic analysis [1-3] to improve data quality and inform targeted interventions. Indeed, they can process thousands of names simultaneously, saving time and resources. However, most of them charge for more than a certain number of requests per month. We recently compared the performance of 4 GDTs and showed that Gender API (Gender-API.com) and NamSor (NamSor Applied Onomastics) were accurate (misclassifications=1.5% and 2.0%, respectively; nonclassifications=0.3% and 0%, respectively) [4].

ChatGPT is a language model developed by OpenAI that is capable of generating human-like text and engaging in natural language conversations [5]. In medicine, ChatGPT can be employed for various purposes, such as answering patient queries and providing information on medical topics, making it a valuable resource for health care professionals and researchers seeking quick access to medical information and support in their work [6,7].

Given the increasing usefulness of GDTs in research, particularly for evaluating gender disparities in medicine, we assessed whether the performance of ChatGPT as a free GDT (version GPT-3.5) could approach that of Gender API and NamSor. We also compared ChatGPT-3.5 with the more advanced GPT-4 version. We hypothesized that ChatGPT, a versatile language model not specifically trained for gender analysis, could achieve gender detection performance comparable to specialized tools and that ChatGPT-4 would perform no better than ChatGPT-3.5.

## Methods

### Database Selection and Data Collection

The methods used in this study are the same as those used in our primary study, which compared the performance of 4 GDTs [4]. We used a database of 6131 physicians practicing in Switzerland, a multilingual and multicultural country with 36% of physicians of foreign origin [4]. The sample consisted of 3085 women (50.3%) and 3046 men (49.7%), with gender determined by self-identification. We used nationalize.io to determine the origin of physicians’ names (Table 1). A total of 88% of names were from French-, English-, Spanish-, Italian-, German-, or Portuguese-speaking countries or from another European country.

We asked ChatGPT-3.5 to determine the gender of 500 physicians at a time, after copying and pasting these lists of first and last names from the database. We ran the analysis twice and also examined ChatGPT-4 to check the “stability” of the responses [8]. The data were collected between September and November 2023.

We constructed a confusion matrix (Table 2): *ff* and *mm* correspond to correct classifications, *mf* and *fm* to misclassifications, and *fu* and *mu* to nonclassifications (ie, gender impossible to determine).

As in other studies [4,9], we calculated 4 performance metrics, namely “errorCoded” (the proportion of misclassifications and nonclassifications), “errorCodedWithoutNA” (the proportion of misclassifications), “naCoded” (the proportion of nonclassifications), and “errorGenderBias” (the direction of bias in gender determination). We used Cohen κ to assess interrater agreement.

**Table 1:** Estimated origin of physicians’ names (N=6131 physicians).

Origin	Count^a^, n (%)
French-speaking country	1679 (32.2)
English-speaking country	751 (14.4)
Spanish-speaking country	404 (7.7)
Asian country^b^	344 (6.6)
Eastern European country	324 (6.2)
Italian-speaking country	288 (5.5)
Western European country^b^	272 (5.2)
Arabic-speaking country	259 (5.0)
German-speaking country	259 (5.0)
Northern European country^b^	220 (4.2)
Southern European country^b^	217 (4.2)
Portuguese-speaking country	198 (3.8)

^a^The total number of physicians does not add to 6131 because of missing values (no assignments for 916 physicians).

^b^If not already classified in another group (eg, in the Arabic-speaking country group for some Asian countries).

**Table 2:** Confusion matrix showing the 6 possible classification outcomes.

	Female (predicted)	Male (predicted)	Unknown (predicted)
Female (actual)	ff	fm	fu
Male (actual)	mf	mm	mu

### Ethical Considerations

Since this study did not involve the collection of personal health–related data, it did not require ethical review per current Swiss law.

## Results

Performance metrics showed high accuracy for ChatGPT-3.5 and ChatGPT-4 in both the first and second rounds (Table 3). The number of misclassifications was low (proportion≤1.5%) and there were no “nonclassifications.” As shown in Table 3, interrater agreement between the first and second rounds (for ChatGPT-3.5 and ChatGPT-4) and between ChatGPT-3.5 and ChatGPT-4 (for the first round) was “almost perfect” (κ>0.97, all *P*s<.001).

**Table 3:** Confusion matrix and performance metrics for ChatGPT-3.5 and ChatGPT-4 (N=6131 physicians).

					Classified as women, n (%)			Classified as men, n (%)			Unclassified, n (%)			Interrater agreement^a^			
														Cohen κ (95% CI)			*P* value
**ChatGPT-3.5**														0.9817 (0.9770-0.9865)^b^			<.001
	**First round^c^**																
		Female physicians (n=3085)	3028 (98.2)			57 (1.8)			0 (0)								
		Male physicians (n=3046)	18 (0.6)			3028 (99.4)			0 (0)								
	**Second round^d^**																
		Female physicians (n=3085)	3030 (98.2)			55 (1.8)			0 (0)								
		Male physicians (n=3046)	28 (0.9)			3018 (99.1)			0 (0)								
**ChatGPT-4**														0.9958 (0.9935-0.9981)^b^			<.001
	**First round^e^**																
		Female physicians (n=3085)	3020 (97.9)			65 (2.1)			0 (0)								
		Male physicians (n=3046)	27 (0.9)			3019 (99.1)			0 (0)								
	**Second round^f^**																
		Female physicians (n=3085)	3020 (97.9)			65 (2.1)			0 (0)								
		Male physicians (n=3046)	26 (0.9)			3020 (99.1)			0 (0)								

^a^Interrater agreement between ChatGPT-3.5 and ChatGPT-4 (for the first round): Cohen κ=0.9768, 95% CI 0.9715-0.9822, *P*<.001.

^b^Interrater agreement between the first and second rounds for each version.

^c^Performance metrics: errorCoded=0.01223, errorCodedWithoutNA=0.01223, naCoded=0, and errorGenderBias=–0.00636.

^d^Performance metrics: errorCoded=0.01354, errorCodedWithoutNA=0.01354, naCoded=0, and errorGenderBias=–0.00440.

^e^Performance metrics: errorCoded=0.01501, errorCodedWithoutNA=0.01501, naCoded=0, and errorGenderBias=–0.00620.

^f^Performance metrics: errorCoded=0.01484, errorCodedWithoutNA=0.01484, naCoded=0, and errorGenderBias=–0.00636.

## Discussion

We used ChatGPT to determine the gender of 6131 physicians practicing in Switzerland and found that the proportion of misclassifications was ≤1.5% for both versions. There were no nonclassifications and gender bias was negligible. Interrater agreement between ChatGPT-3.5 and ChatGPT-4 was “almost perfect.”

These results are relatively similar to those found in our primary study for Gender API and NamSor (errorCoded=0.0181 and 0.0202, errorCodedWithoutNA=0.0147 and 0.0202, naCoded=0.0034 and 0, errorGenderBias=–0.0072 and 0.0026) [4]. They are slightly better than those of another study published in 2018, which compared 5 GDTs, including Gender API and NamSor [9]. These results suggest that ChatGPT can accurately determine the gender of individuals using their first and last names. The disadvantage of ChatGPT compared to Gender API and NamSor is that the database cannot be uploaded directly into ChatGPT (eg, as an Excel or CSV file).

Both ChatGPT-3.5 and ChatGPT-4 exhibit high accuracy in gender detection, with no significant superiority observed in ChatGPT-4 over ChatGPT-3.5. This underscores the robustness of ChatGPT in gender prediction across different versions. Our short study has 2 main limitations. Given the estimated origin of physicians’ names, the results of the study can probably be generalized to most Western countries but not necessarily to Asian or Middle Eastern countries. GDTs are often less accurate with names from these countries [9,10]. In addition, GDTs oversimplify the concept of gender by dichotomizing individuals into male or female.

## Abbreviations

GDT: gender detection tool

## Data Availability

The data associated with this article are available in the Open Science Framework [11].
